# βII spectrin (SPTBN1): biological function and clinical potential in cancer and other diseases

**DOI:** 10.7150/ijbs.52375

**Published:** 2021-01-01

**Authors:** Panyu Yang, Yanyan Yang, Pin Sun, Yu Tian, Fang Gao, Chen Wang, Tingyu Zong, Min Li, Ying Zhang, Tao Yu, Zhirong Jiang

**Affiliations:** 1 Department of Cardiac Ultrasound, The Affiliated Hospital of Qingdao University, Qingdao 266000, China.; 2 Department of Immunology, Basic Medicine School, Qingdao University, No. 308 Ningxia Road, Qingdao 266071, People's Republic of China.; 3 Institute for Translational Medicine, The Affiliated Hospital of Qingdao University, No. 38 Dengzhou Road, Qingdao 266021, People's Republic of China.; 4 Department of Physical Medicine and Rehabiliation, The Affiliated Hospital of Qingdao University, Qingdao, Shandong Province, China.

**Keywords:** βII Spectrin, proliferation, cancers, heart, development, TGF-β

## Abstract

βII spectrin, the most common isoform of non-erythrocyte spectrin, is a cytoskeleton protein present in all nucleated cells. Interestingly, βII spectrin is essential for the development of various organs such as nerve, epithelium, inner ear, liver and heart. The functions of βII spectrin include not only establishing and maintaining the cell structure but also regulating a variety of cellular functions, such as cell apoptosis, cell adhesion, cell spreading and cell cycle regulation. Notably, βII spectrin dysfunction is associated with embryonic lethality and the DNA damage response. More recently, the detection of altered βII spectrin expression in tumors indicated that βII spectrin might be involved in the development and progression of cancer. Its mutations and disorders could result in developmental disabilities and various diseases. The versatile roles of βII spectrin in disease have been examined in an increasing number of studies; nonetheless, the exact mechanisms of βII spectrin are still poorly understood. Thus, we summarize the structural features and biological roles of βII spectrin and discuss its molecular mechanisms and functions in development, homeostasis, regeneration and differentiation. This review highlight the potential effects of βII spectrin dysfunction in cancer and other diseases, outstanding questions for the future investigation of therapeutic targets. The investigation of the regulatory mechanism of βII spectrin signal inactivation and recovery may bring hope for future therapy of related diseases.

## Introduction

Spectrin, first discovered in erythroid cells by Marchesi and Steersand in 1968 1, is expressed in all metazoan cells and highly conserved across different species 2. Spectrin forms an extensive filamentous intracellular network that acts as a cytoplasmic scaffold for proteins 3. Due to the high affinity between the α and β chains, spectrin exists mainly as heterotetramers rather than autonomous α or β subunits 4. Spectrin is expressed in numerous tissues and cells. A protein similar to spectrin was identified in neurons and originally named fodrin 5. Interestingly, it was later discovered that fodrin is a common spectrin in most cells 3, 6. The spectrin-based membrane skeleton is essential for the mechanical stability and elasticity of red blood cells. It mainly contributes to membrane integrity, organization and trafficking 7. Defects in the erythrocyte membrane skeleton can lead to a variety of hemolytic anemias 8. In most cells, spectrin is involved in determining cell shape and maintaining cell flexibility, intercellular contact, cell polarity, and proliferation 4. Although spectrin has also been found in the nuclei of non-erythrocytes, its function remains to be elucidated.

In mammals, spectrin consists of modular structures of α and β subunits expressed by seven genes (αI, αII, βI, βII, βIII, βIV, and βV). The αI and αII spectrin isoforms are encoded by SPTA1 and SPTAN1, respectively. Conventional β spectrin isoforms (βI‐IV) consist of diverse subunits that are encoded by four different genes, SPTB, SPTBN1, SPTBN2, and SPTBN4, as well as SPTBN5 encodes a large βV-spectrin 9.

βII spectrin (β2-Spectrin/SPTBN1/β2SP/ELF, gene SPTBN1), a dynamic intracellular protein usually located on the medial membrane of the cell. Notably, the versatile role of βII spectrin in disease has been reported in an increasing numbers of studies, and βII spectrin has been implicated in diseases such as cancer, neurological disorders, cardiovascular diseases, osteoporosis, and hearing loss 2, 10-13. Strikingly, growing evidence associates βII spectrin with various cancers, and expression and function vary between different tumor stages or types of βII spectrin 10. Moreover, the most serious effect was observed in mice deficient in βII spectrin, which caused embryonic lethality 14. In recent years, βII spectrin has been shown to be responsible for multiple diseases, highlighting the critical role of βII spectrin in metabolism.

In this review, we introduce the structure of βII spectrin and discuss the molecular mechanisms associated with its diverse protein domains, particularly the role of βII spectrin in regulating proliferation, controlling cell adhesion and cell cycle progression, and maintaining genomic stability. Furthermore, we highlight the implications of βII spectrin deficiency or dysfunction in development and human diseases.

## Structural insights into βII spectrin

Spectrin is a complex of two similar peptides called the α and β subunits 1, 15. The α and β subunits are assembled side by side in an antiparallel manner to form rod-shaped αβ dimers. Subsequently, the N-terminus of each α subunit of the dimer and the C-terminus of each β subunit interact to form a self-associated head-to-head tetramer 4.

Spectrin, which has many structural motifs and is highly modular, is characterized by the presence of spectrin repeats (SRs, composed of 106 contiguous amino acid sequence motifs) 16, actin-binding domains (ABDs) (calponin homology (CH) domains), EF-hands, pleckstrin homology (PH) domains, Src homology 3 (SH3) domains and many other signaling domains and motifs, and its structure contributes to the multiple functions of spectrin 17, 18.

**Figure 1:**
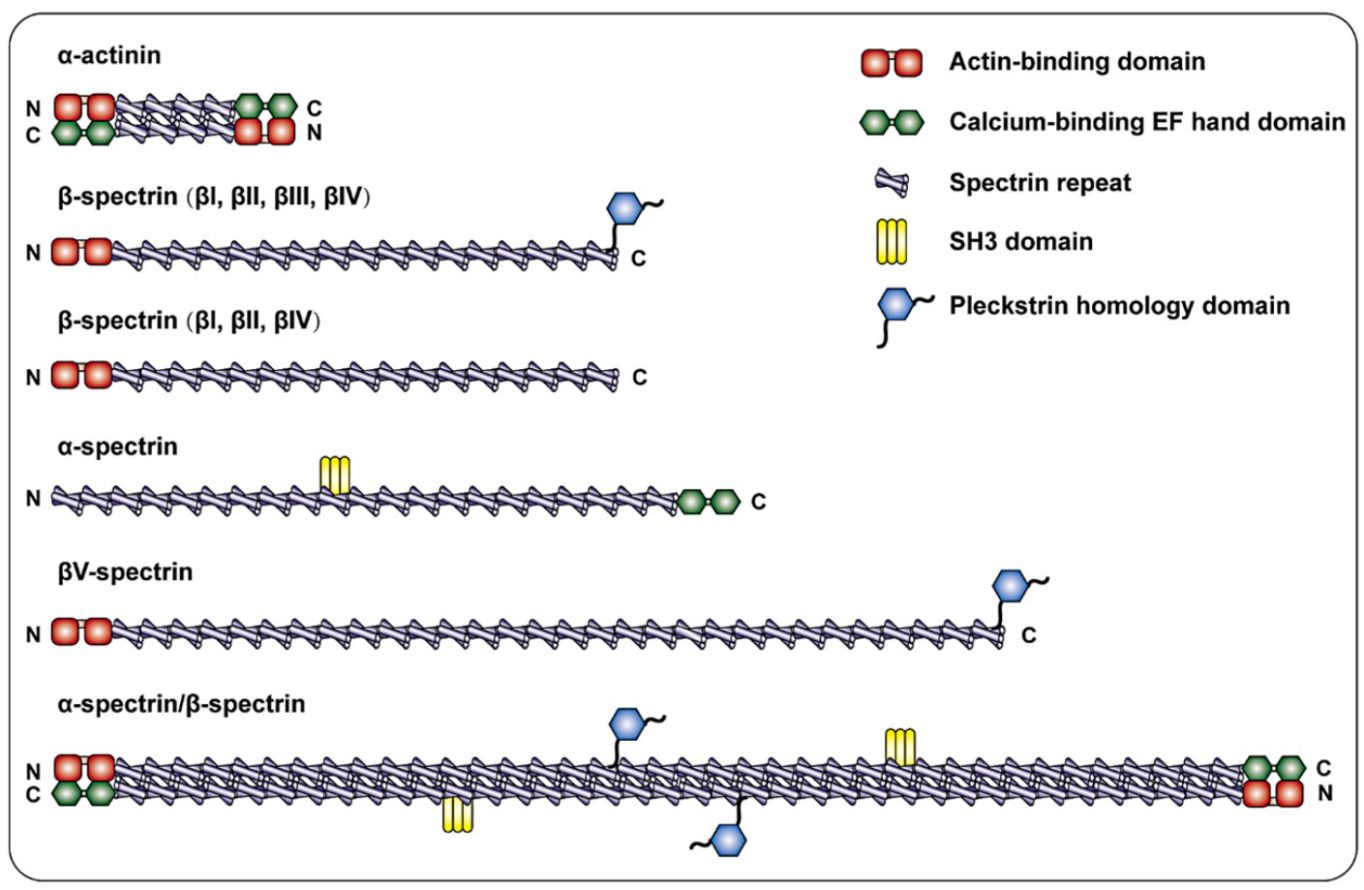
** Schematic diagram of the spectrin family and α-actinin.** α-actinin consists of two actin-binding domains (ABD) CH1 and CH2, four spectrin repeats, and a COOH-terminal EF hand domain to form a single chain, and the two single chains are antiparallel to form dimerization body. β-spectrin is composed of two CH1 and CH2 domains at the NH_2_- terminal followed by 17 spectrin repeats. β I, β II, and β IV spectrins are cleaved into a long COOH-terminal containing PH domain or a short COOH-terminal not containing PH domain. In addition to the spectrin repeat sequence, α-Spectrin also includes the SH3 site of domain 10 and the EF hand domain at the end of COOH. β-H spectrin is similar in structure to other β- spectrin, and is characterized by containing more spectrin repeats.

The most common non-erythrocyte spectrin isoform is βII spectrin, which is present in all nucleated cells and forms a large polymer complex with ankyrin and actin 19. According to Broderick and his colleague, the evolutionary process from α-actinin to αβ-spectrin was complex. The addition of SRs extended α-actin, which led to the replication of αβ ancestors and subsequent separation into β and α chains. Among them, β-spectrin corresponds to the N-terminus of the ancestor α-actinin, and α-spectrin corresponds to the C-terminus. Therefore, β-spectrin is composed of the CH1 and CH2 domains followed by SRs 20. Specifically, the NH2 terminal ABDs of β-Spectrin consists of a pair of CH domains 21, 22. The presence of the ABDs allows β spectrin to interact with F-actin in a variety of different cellular situations. Connected to the ABDs of the β subunit are 17 consecutive triple helix motifs that terminate in a carboxyl region 23. Additionally, βII spectrin has a variety of splice variants, including "long" and "short" C-terminal regions (βIIΣ1 and βIIΣ2) 24, the long COOH terminal region of β-spectrin has a PH domain 25. α-Spectrin has 22 domains: domains 1-9 and 11-21 are composed of SR, domain 10 is the SH3 site 26, and the COOH-terminus is the EF-hand 27. The β-H spectrin contains 30 SR sequences (Figure 1). The above depicts the structure of spectrin in mammals.

## Function of βII spectrin

### βII spectrin regulates cell proliferation

βII spectrin has important functions in liver cell proliferation and liver regeneration. Hepatocyte proliferation was inhibited after transforming growth factor β1 (TGF-β1) stimulation 28. Insulin is a hepatocellular mitogen. Treatment of AML-12 cells (normal mouse hepatocytes) with insulin (100 nM) activates the Phosphoinositide 3-kinase (PI3K)/Protein kinase B (Akt) pathway, which increases phosphor-Akt expression and nuclear translocation of Smad2, 3 and 4. LY294002 is an inhibitor of PI3K that can completely block Akt phosphorylation and can also significantly reduce βII spectrin, Smad3 and 4 in the nucleus 28. Strikingly, this study indicated that TGF-β/Smad pathway can be activated by PI3K/pAkt signalling, which is downregulated by activating of TGF-β/Smad pathway 28. In hepatocyte proliferation, TGF-β/Smad and PI3K/AKT signaling pathways interact to regulate proliferation, and βII spectrin is involved in these processes. Hepatocyte dedifferentiation is a crucial cellular event in the development of liver cancer 29. A related study showed that βII spectrin could promote the differentiation and inhibit the growth of hepatocellular carcinoma (HCC) cells in vitro 30. These findings indicate that βII spectrin is vital in liver cell proliferation and promotes liver regeneration after liver resection in mice. βII spectrin is essential for the survival of liver cells and endothelial cells. Strikingly, the loss of βII spectrin may lead to angiogenesis stimulation 31. Inadequate βII spectrin led to dysregulated hepatocyte proliferation as well as abnormal and uncontrolled proliferation of yolk sac vascular endothelial cells 32. βII spectrin is also necessary for embryonic liver development. βII spectrin mutant embryos (βII spectrin^-/-^) had an abnormal liver structure and enhanced hepatocyte proliferation capacity 28. Moreover, another study showed that cluster of differentiation 133 (CD133^+^) liver stem cells (LSCs) in βII spectrin^+/-^ mice treated with IL6 were highly proliferative 33. All of the above results indicate that the effects of the loss of βII spectrin are related to the regulation of cell proliferation.

### βII spectrin regulates differentiation and development

**Figure 2:**
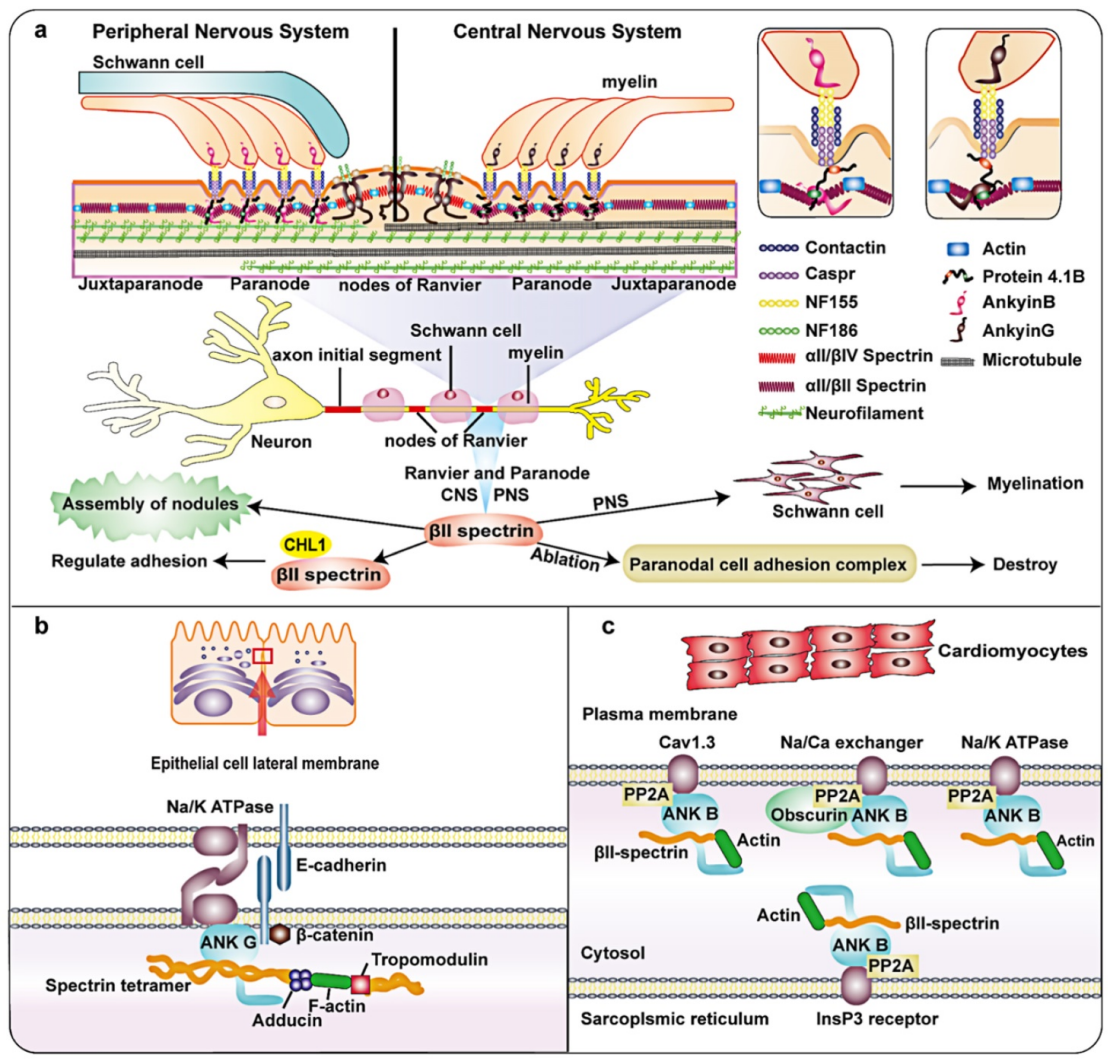
** Schematic diagram and partial functions of βII spectrin in nerve fibers, epithelial cells and the heart.** a: Axons are composed of parallel bundles of microtubules that act as a structural skeleton. Myelin sheaths are produced by oligodendrocytes in the central nervous system (CNS) and Schwann cells in the peripheral nervous system (PNS). Myelin axons can be subdivided into Ranvier nodes, paranode and adjacent paranodes. The paranodes contain axon cell adhesion molecules (CAM), such as neurofascin 155 (NF155) on the glial membrane and a complex of Caspr and contactin on the axon. In addition, there are Protein 4.1B, αII/βII spectrin tetramer, and ankyrin G in the CNS and ankyrin B in PNS. Ranvier nodes include neurofascin 186 (NF-186), αII/βIV Spectrin, and ankyrin G. βII spectrin is essential for microtubule stability and glial complexes. It is critical in the assembly of the axon initial segment (AIS), Ranvier and paranode. b: In the lateral epithelial membrane module, ankyrin-G, E-cadherin and Na^+^/K^ +^ ATPase are combined. The F-actin bridge bound by tropomodulin is connected to α/γ adducin. βII spectrin, adducin and ankyrin-G directly combine to form a complex. c: In the heart, βII spectrin is involved in the localization and stability of transporters. It forms a complex with ankyrin B, which can target type 2A protein phosphatase (PP2A) and plays a role in transporters such as Na/K-ATPase, Cav1.3, InsP3R and Na/Ga exchanger. In addition, βII spectrin also binds to Actin.

Axons are important structures that connect the nervous system. They consist of parallel microtubule bundles, which act as a structural skeleton, surrounded by regularly spaced actin loops called periodic membrane skeletons. Myelin sheaths are produced by oligodendrocytes in the central nervous system (CNS) and Schwann cells in the peripheral nervous system (PNS). βII spectrin is relatively abundant in the nervous system and has important functions 3. Myelinated axons can be subdivided into some domains such as Ranvier nodes, paranodes and juxtaparanodes (Figure 2a) 34. Each of these domains contains a unique protein complex consisting of glial and axon cell adhesion molecules, ion channels, and cytoskeletal/scaffold proteins. Axon complexes are anchored to the axon cytoskeleton and crucial for effective nodes formation 35. The paranode contains cell adhesion molecules: neurofascin 155 on the glial membrane, Caspr and contactin 36. Protein 4.1B has been identified at the paranode 37. In addition, the paranode also include αII/βII spectrin tetramers. Ranvier node's cell adhesion molecule is different from paranode. During the formation of myelin in the peripheral nervous system (PNS), Schwann cell proteins direct the aggregation of axonal cell adhesion molecules glialneurofascin 186 (NF-186) at the Ranvier node 38. When this connection breaks, changes in both the adaptor protein 4.1B and the cytoskeleton protein βII spectrin in the axon lead to misalignment, and the /efficiency of Ranvier node assembly is greatly reduced 39. Notably, high-density Na^+^ channels on the nodes of Ranvier are necessary for the rapid and effective propagation of action potentials in myelinated axons 40, 41. A recent study showed that Ranvier nodes of Na^+^ channels that depend on paranodes require axonal βII spectrin, which is concentrated near the paranodes and juxtaparanodes 42, 43. Previous studies have shown that βII spectrin and cyclic actin loops are essential for microtubule stability 44. Silencing AnkyrinB, αII-spectrin, or βII spectrin expression disrupted the axon initial segment (AIS) in mice and prevented AIS assembly 45.

Plasma membrane-associated structural proteins promote axon stability and efficient organelle transport. A recent study in which βII spectrin was knocked down in mouse neural progenitor cells showed that βII spectrin is crucial to axonal transport of transmitters in the mouse brain 46, 47. βII spectrin and AnkyrinB promote axonal transport through different mechanisms 46. βII spectrin without ankyrin-binding activity restores transport, and axonal transport in mice lacking βII spectrin and AnkyrinB was more severely compromised than that in mice lacking either protein alone 46. Consistently, these results show the versatile role of βII spectrin in axonal transport.

αII Spectrin and βII spectrin occupy polarized positions at the axon contact site in premyelinating Schwann cells. In cultured Schwann cells, silencing of βII spectrin could inhibit myelination 48. Observation of conditional knockout mice lacking glial βII spetrin revealed nodes and paranodes have changed during CNS development 49.

Altogether, the versatile roles of βII spectrin in regulating axon stability, axonal transport, axonal Ranvier node assembly and neurite growth have been demonstrated in increasing numbers of studies.

The sophisticated process of epithelial morphogenesis results in typical epithelial cells with unique protein distribution patterns, enabling them to perform various physiological functions 50. One study indicated that reduced expression of βII spectrinand splicing recruitment play a vital role in stabilization of the connection between the pancreas and colonic epithelium and the regulation of connection remodeling 51. Consistent with this, the depletion of βII spectrin further weakened reconstruction of the cell barrier next to human intestinal epithelial cells and inhibited the formation of important structures in human bronchial epithelial (HBE) cells 51. During the formation of columnar human intestinal cell apical domains, the levels of actin-binding proteins in the terminal network increased in vitro and in vivo. In addition, βII spectrin form a complex dependent on the actin-binding protein that connects F-actin to the top of the microtubule network during epithelial cell morphogenesis 52. The lateral membrane of epithelial cells is an important part of cell adhesion and salt and water homeostasis. As one of the most abundant specialized membrane domains in biology, it has important physiological significance. Studies have shown that the lateral membrane of epithelial cells may be similar to the membrane skeleton of spectrin, with spectrin and ankyrin isoforms positioned at the site of contact between cells (Figure 2b) 53. Some research results have explained the possible related mechanisms. A study on the lateral membrane of epithelial cells showed that adducin adopts a new mechanism to regulate overall characteristics of the outer membrane of the bronchial epithelium by interacting with βII spectrin, which stabilizes the HBE at the lateral membranes of cells 54. In addition, previous findings and recent studies have shown that 4.1N, βII spectrin and ankyrin G are structural components of the lateral membrane backbone 55, 56.

The above research indicates that βII spectrin is highly significant in the assembly of fully functional lateral membranes, the regulation of connection remodeling and stabilization of the connection with the epithelium.

Previous studies have shown that αII-spectrin is involved in regulating the cytoplasmic actin network of cardiomyocytes 57. In recent years, several lines of evidence have demonstrated that βII spectrin, as part of the cytoskeleton, in the heart is critical in maintaining normal development of the heart during embryogenesis. In the heart, βII spectrin and ankyrin B form a complex. Ankyrin B can target type 2A protein phosphatase (PP2A). The complex formed by the interaction of βII spectrin and ankyrin-B plays an important role in the positioning and stability of ion channels and transporters (such as Na/K-ATPase, Cav1.3, InsP3R and NCX) (Figure 2c) 58. Deletion of βII spectrin during cardiac development led to cardiac developmental defects, accompanied by cardiac muscle cell differentiation and abnormal cytoskeleton formation. Consistent with this, muscle differentiation, like the expression of dystrophin, homeobox protein Nkx-2 (Nkx2.5), and alpha-smooth muscle actin (α-SMA), was markedly decreased. The mechanism involved in the destruction of βII spectrin reduces Smad signaling in the TGF-β signaling pathway. Studies have shown that the loss of βII spectrin can cause heart wall thickening failure and decreased cell density in the trabecular layer of the mouse heart, leading to heart developmental defects 59. These findings indicate that βII spectrin is essential for cardiomyocyte differentiation and cardiac development.

Inner ear hair cells (HCs) is crucial in hearing and can detect sound. This process is achieved by the deflection of mechanosensory stereocilia 60. Recent studies indicated that spectrin in HCs is essential in deafness and hearing development. In the early stages of development, HC polarity is destroyed in the loss of βII spectrin, and the degree of destruction becomes greatest in the later stages of development, which eventually causes HCs degeneration beginning approximately 2 weeks after birth 13. In addition, in different animal models of hearing loss, the spectrin loop was associated with auditory ability 13. These results demonstrate the critical role of spectrin in auditory development in the epidermal plate.

Collectively, βII spectrin is essential for the development of organs such as nerve, epithelium, inner ear and heart. The absence of βII spectrin causes changes in related structures, which in turn affect the function of various organ systems. Therefore, its importance in development could be considered as a potential therapeutic target in treatment of related organs in future.

### βII spectrin regulates cell cycle progression

**Figure 3:**
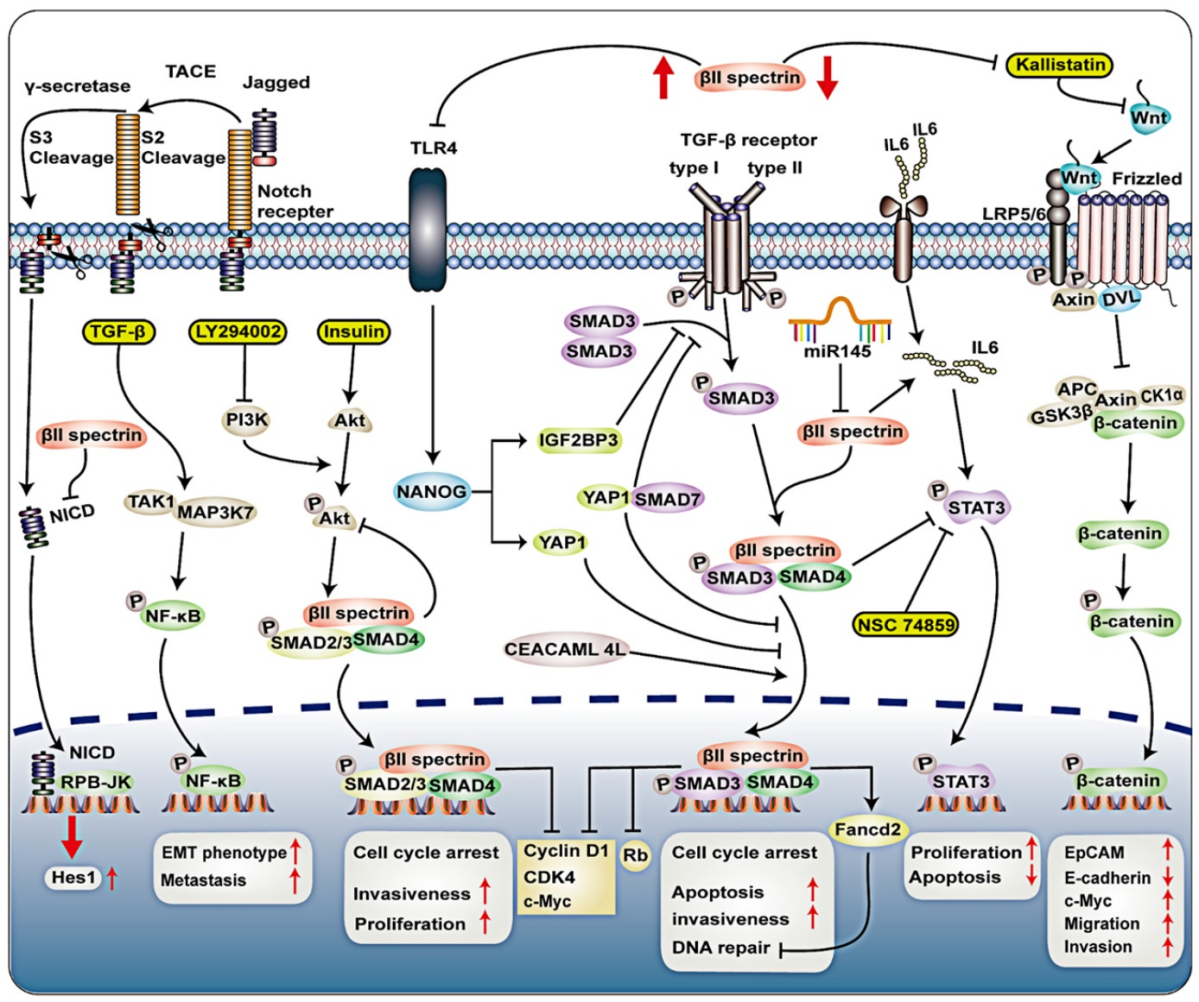
** Signal transduction of βII spectrin in tumor.** Loss of Smad4 and βII spectrin was observed in Barrett's esophagus and adenocarcinoma tissue sections. The absence of βII spectrin/Smad4 will interrupt TGF-β signaling, which may activate Notch signaling through the Notch signaling molecule Hes-1. In CD133^+^ LSCs, TAK1-NFκB signal and TGF-β signaling pathway activated kinase 1 (TAK1; MAP3K7), leading to increased production of metastatic CSCs. Treatment of AML-12 cells with insulin (100 nM) activates the PI3K/Akt pathway, which increases phosphor-Akt expression and nuclear translocation of Smad2, 3 and 4. LY294002 is an inhibitor of PI3K that can completely block Akt phosphorylation and can also reduce βII spectrin, Smad 3 and 4 in the nucleus. βII spectrin is involved in hepatocyte proliferation through the interaction of TGF-β/Smad and PI3K/AKT signalling. PI3K/pAkt signalling activated TGF-β/Smad pathway, and activation of TGF-β/Smad pathway downregulated PI3K/pAkt signallings. CEACAM1-4L can interact with βII spectrin, promote nuclear translocation of Smad3 in advanced HCC. TGF-β signaling pathway upregulates Fancd2 expression mediated by βII spectrin and Smad3, thereby conferring genomic stability. In hepatocellular carcinoma (HCC), TLR4 activates NANOG, NANOG induces IGF2BP3 and YAP1, IGF2BP3 blocks the phosphorylation of SMAD3, YAP1 blocks the nuclear translocation of p-SMAD3. IGF2BP3-mediated activation of AKT phosphorylates YAP1, enhancing YAP1's ability to block nuclear translocation of p-SMAD3. YAP1 may interact with SMAD7, and enhance the inhibitory activity and nuclear translocation of SMAD7 on TGF-β/SMAD3 signaling. By drinking alcohol, inhibiting βII spectrin in liver cancer cells can significantly increase the expression of TLR4. IL6 can activate transcriptional activator 3 (STAT3), βII spectrin prevented the development of HCC by downregulating the expression of signal transducer and STAT3. In pancreatic cancer cell lines, miR-145 induces downregulation of βII spectrin.

In HCC cells, βII spectrin can regulate the G1/S cell cycle transition through the TGF-β signaling pathway (Figure 3). During this regulatory process, the expression of Cyclin-dependent kinase 4 (CDK4), cyclin D1 and retinoblastoma protein (Rb) and other G1/S cell cycle checkpoint-related proteins was reduced following βII spectrin overexpression in HCC 31. βII spectrin plays a crucial role in the translocation of Smads, and its loss caused G1/S phase transition due to the activation of cyclin D1/CDK4 in HCC 31. Consistently, when βII spectrin was removed from cardiomyocytes, mitotic activity was reduced 59. Increasing expression of the proapoptotic protein Bax and the cleavage of caspase-7 also indicated cardiomyocyte apoptosis and dysregulation of the cell cycle 59, both of which are closely related to the lack of βII spectrin, proving that its loss causes cell cycle arrest.

### βII spectrin regulates cell adhesion and spreading

Decreased SPTBN1 expression promoted sphere formation, tumor development and aggressive phenotypes in HCC cells 61. Zhi et al. showed that epithelial cell adhesion molecule-positive (EpCAM (+)) cells and integral EpCAM expression were significantly increased in SPTBN1 (+/-) mice compared to wild-type mice 61. Carcinoembryonic antigen-related cell adhesion molecule 1 (CEACAM1-4L) with a long cytoplasmic domain has a tumor suppressive effect and is down-regulated in HCC 62. In contrast, data from an in vitro study demonstrated that CEACAM1-4L can regulate the TGF-β/Smad signaling pathway, interact with βII spectrin, promote nuclear translocation of Smad3 in advanced HCC (Figure 3) 63. Overexpression of CEACAM1-4L enhances the invasiveness of HCC cells. Nonetheless, clarification of the mechanism by which the overexpression of βII spectrin in liver cancer promotes the invasion of liver cancer cells needs further research.

Studies have shown that the growth of close homolog of L1 (CHL1)-dependent neurites requires regulation of the endocytosis of the Ca2^+^-dependent CHL1-βII spectrin complex and CHL1, ligand induction, and lipid raft-dependent CHL1 adhesion regulation (Figure 1) 64.

Proteomic analysis of the lack of breast cancer sensitivity gene 1 (BRCA1) in epithelial ovarian cancer (EOC) showed a 2.5-fold reduction in βII spectrin expression, which was related to regulation of cell migration and invasion 65. As a component of the cytoskeleton, βII spectrin has a certain regulatory effect on the cytoskeleton/actin cell adhesion in EOC. However, the expression of endometrial side population cells in patients with early and advanced disease and their underlying regulatory mechanisms require more research.

The interaction of subperitoneal fibroblasts with cancer cells in peritoneal tissues showed that validated cases with overexpression of βII spectrin during tumor progression had a poor prognosis. Overexpression of βII spectrin may promote tumor progression and metastasis through some means (such as biological or mechanical means) 66.

The above results indicate that the overexpression of βII spectrin is related to enhanced tumor aggressiveness and that its deletion is also closely related to cell adhesion and migration.

## βII spectrin maintains genomic stability

The stability of the genome is crucial to the development of diseases, especially cancer. Previous studies suggest that TGF-β1 is a key regulator of genomic integrity 67. A recent study by Chen et al. demonstrated the major role of the TGF-β/Smad3 adapter βII spectrin in maintaining genomic stability after alcohol-induced DNA damage (Figure 3) 68. It has been suggested that the TGF-β signaling pathway upregulates Fancd2 (a core component of the Fanconi anemia complex) expression mediated by βII spectrin and Smad3, thereby conferring genomic stability and proper DNA repair. βII spectrin deficiency caused genomic instability and increased sensitivity to DNA damage. βII spectrin-deficient cells showed DNA crosslinking and DNA double-strand breaks after injury and stimulation 68. DNA double-strand break repair defects could be repaired by ectopic Fancd2 expression. Further studies on DNA damage repair pathways confirmed that deletion of βII spectrin led to a defect in homologous recombination DNA repair 68. Overall, this indicates that βII spectrin is beneficial to DNA repair through the TGF-β pathway.

## The involvement of βII spectrin in cancer

**Figure 4:**
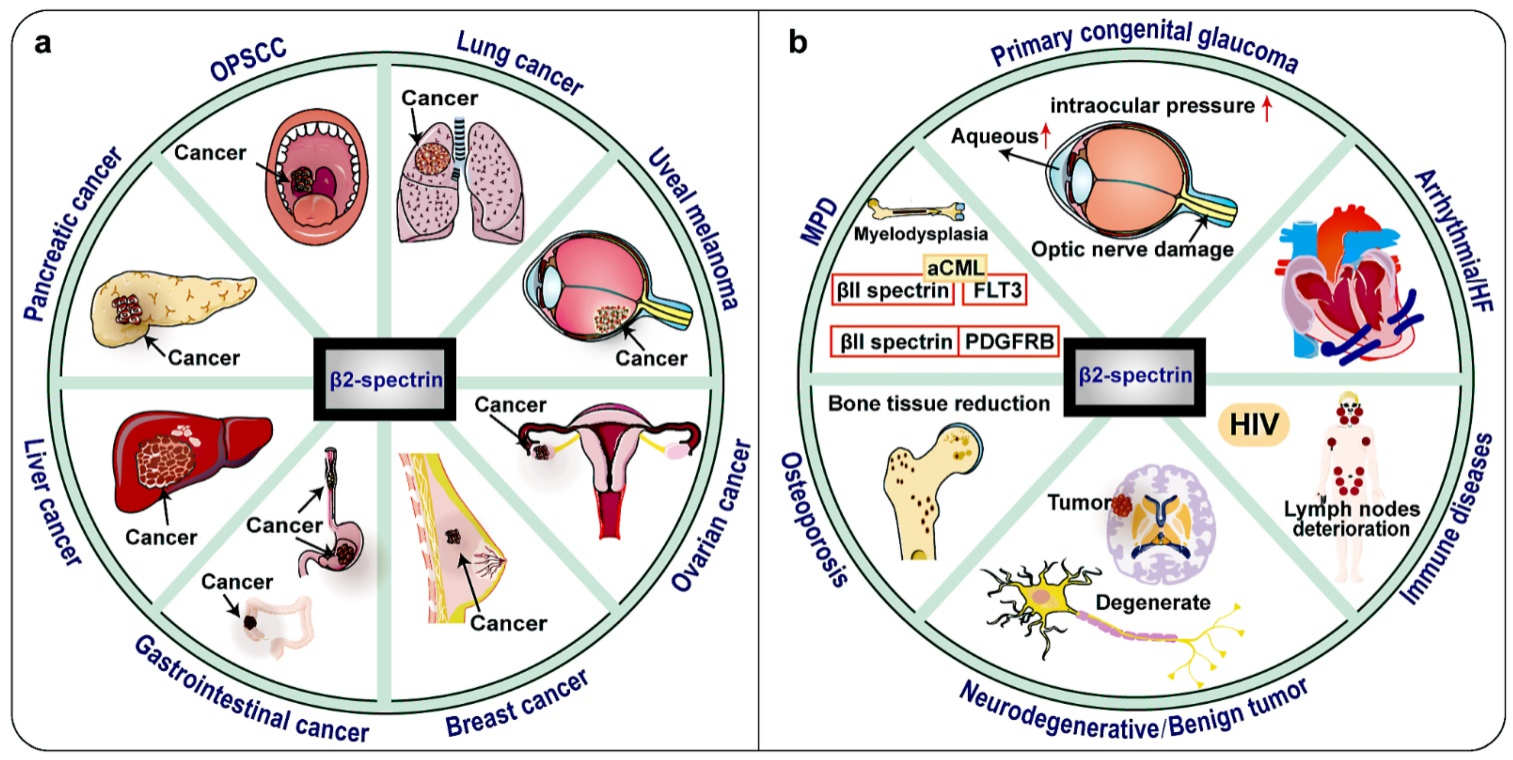
** Tumors and other diseases related to βII spectrin.** βII spectrin is associated with a variety of tumors and has a tumor suppressing effect, and its expression is down-regulated in most tumors. In some hepatocellular carcinomas (HCC), the expression of βII spectrin is up-regulated and the invasiveness is also increased. In HIV, βII spectrin is related to the sensitivity of macrophages to HIV virus, and its expression is up-regulated. SPTBN1 is found to be fused with other genes in lung cancer, ovarian cancer, atypical chronic myeloid leukemia (aCML) and myeloproliferative disease (MPD). βII spectrin is mutated in oropharyngeal squamous cell carcinomas (OPSCC), Osteoporosis and Neurodegenerative diseases. No changes in βII spectrin expression have been found in heart failure cases.

Notably, clinical research data shows that the expression of βII spectrin is significantly reduced in malignant tumors such as most human HCC, lung cancer, digestive tract cancer, pancreatic cancer and so on (Fig. 4, Table 1) 30, 69-71. TGF-β/Smad, Wnt/β-catenin, Notch, NF-κB, IL-6/transcription 3 (STAT3) and other signaling pathways are now known to be important in the relationship between βII spectrin and cancer 10. This raises the possibility that βII spectrin can be used as a diagnostic biomarker and for a molecular assessment of prognosis.

### βII spectrin in liver cancer

Most patients with HCC are already at an advanced stage when diagnosed with liver cancer, so their prognosis is poor 72. Therefore, there is an urgent need to demonstrate the molecular mechanisms of liver cancer progression, as these studies are the basis for promoting the development in biomarkers and therapies for this disease.

Forty percent of βII spectrin (+/-) mice developed spontaneous HCC, and the expression of βII spectrin was significantly reduced in most human HCC cases 61. Decreased expression of βII spectrin is associated with shortened HCC survival 32, suggesting that this protein has the ability to inhibit tumors 73. Lin et al*.* found that βII spectrin prevented the development of HCC by downregulating the expression of signal transducer and transcriptional activator 3 (STAT3) 74. Loss of βII Spectrin promotes Wnt signaling activation through down-regulation of Kallistatin, increased expression of EpCAM and c-Myc, and decreased expression of E-cadherin, which are downstream genes of Wnt/β-catenin signaling 61, 75. Kallistatin is a Wnt antagonist that binds to low density lipoprotein receptor-related protein 6 (LRP6). The main component of β-catenin is strictly regulated by the destruction complex, which consists of the scaffold protein AXIN, adenomatous polyposis (APC), and the kinases glycogen synthase kinase 3α/β (GSK3) and casein kinase (CK1α) 76. Under active conditions, Wnt ligands bind to frizzled (FZD) receptors and LRP5/6 receptors. Next, scattered scaffold proteins (DVL) and AXIN are recruited to the membrane. The chelation and degradation of AXIN leads to the disintegration of β-catenin destruction complex 77, and then unphosphorylated β-catenin accumulates in the cytoplasm, and active β-catenin translocates to the nucleus 78. Moreover, the loss of SPTBN1 and Kallistatin protein was associated with a higher recurrence rate and increased tumor migration and invasion (Figure 3) 61. Previous studies have demonstrated that toll-like receptor 4 (TLR4) and TGF-β pathways have antagonistic effects in HCC 79. Knocking down β2-Spectrin in liver cancer cells can significantly increase the expression of TLR4 and enhance the self-renewal and tumorigenic activity of the cells. TLR4 activates NANOG, NANOG induces IGF2BP3 and Yes-associated protein 1 (YAP1), IGF2BP3 blocks the phosphorylation of SMAD3, YAP1 blocks the nuclear translocation of p-SMAD3, thereby blocking the TGF-β pathway 79, IGF2BP3 and YAP1 are Tumor-initiating stem-like cells (TICs) oncogene 80. In addition, IGF2BP3-mediated activation of AKT phosphorylates YAP1, thereby enhancing YAP1's ability to block nuclear translocation of p-SMAD3 79. YAP1 may interact with SMAD7, and enhance the inhibitory activity and nuclear translocation of SMAD7 on TGF-β/SMAD3 signaling 62. By drinking alcohol, inhibiting βII-Spectrin in liver cancer cells can significantly increase the expression of TLR4, and enhance the self-renewal and tumorigenic activity of cells (Figure 3) 79. Altogether, these data suggest that βII spectrin plays a tumor-suppressive role that is executed through diverse signaling pathways in HCC.

Nonetheless, one study demonstrated that βII spectrin was overexpressed in a highly metastatic HCC cell line, was highly expressed at the fragment level in human HCC cell lines 81. The mechanism by which overexpression of βII spectrin in liver cancer accelerated the invasion of liver cancer cells needs further research. Biomarker-driven targeted therapies for HCC in the future may also include DNA repair pathways and, along with other signal transduction pathways, may alter the course of this deadly cancer.

### βII spectrin in pancreatic cancer

The treatment of most pancreatic cancer patients with chemotherapy is difficult because these patients are usually diagnosed with metastatic advanced pancreatic cancer. Therefore, the need to better understand the molecular mechanisms of pancreatic tumorigenesis and test new chemopreventive and chemotherapeutic compounds to establish practical methods for early detection remains.

Low expression of the βII spectrin protein indicates a worse prognosis, and decreased expression of βII spectrin was related to a shorter survival time in patients with pancreatic cancer, suggesting that the protein has a tumor-suppressive function, as shown in other malignant tumors of the gastrointestinal tract 73, 82. In recent years, the role of non-coding RNA in disease has been extensively studied 83-92. Pancreatic cancer cell line-based miRNA analysis and quantitative proteomics analysis revealed that miR-145 induced the downregulation of some protein linked to cancer, including βII spectrin (Figure 3) 70. This study revealed previously unreported miR-145 targets and provided new insights into the tumor-suppressive mechanisms of βII spectrin. βII spectrin(+/-) mice serve as an important animal model for lethal gastrointestinal and pancreatic cancers 93. In short, βII spectrin is closely related to the development and prognosis of pancreatic cancer, but its specific regulatory mechanism in pancreatic cancer needs further research.

### βII spectrin in colorectal cancer, esophageal cancer and gastric cancer

Colorectal inflammation may be the basis of tumor occurrence and progression 94, 95. In automated immunohistochemistry assay-positive colorectal cancer (CRC) cases, target gene enrichment and sequencing showed that Anaplastic lymphoma kinase (ALK) was fused with a new partner, SPTBN1. The authors concluded that this new SPTBN1-ALK fusion gene and other ALK fusion genes could be potential targets for anti-ALK therapy 96, 97. Therefore, SPTBN1 has an important regulatory role in CRC that is mainly related to its antitumor effect.

The loss of Smad4 and β2 spectrin was observed in Barrett's esophagus and adenocarcinoma tissue sections 71. This study indicates for the first time that βII spectrin is important in the loss of TGF-β signaling pathway and activation of Notch signaling pathway in esophageal adenocarcinoma. The absence of βII spectrin/Smad4 will interrupt TGF-β signaling, which may activate Notch signaling through the Notch signaling molecule Hes-1 (Figure 3). The TGF-β pathway is necessary for stem cell differentiation, but Notch maintains the undifferentiated phenotype of stem cells 98. The interaction between TGF-β and Notch pathway is crucial for the transformation of esophageal stem cells. Therefore, restoring abnormal TGF-β signaling function or inhibiting Notch signaling may become a new treatment for esophageal adenocarcinoma.

βII spectrin is critical in the development of gastric cancer through the TGF-β signaling pathway, inhibiting tumors and regulating gastrointestinal epithelial cell adhesion 99, 100. Study on gastric cancer demonstrates that βII spectrin^+/-^/smad4^+/-^ mice are important model of gastric cancer 101. βII spectrin can inhibit the occurrence of gastric cancer. βII spectrin loss-mediated disruption of TGF-β signaling, which leads to abnormal cell cycle regulation and promotes faster entry into the S phase 101.

### βII spectrin in lung cancer

In some lung cancer cell lines, βII spectrin expression is insufficient, and CDK4 expression increases 69. It shows that the role of βII spectrin in lung cancer is similar to other tumors. One report described a rare case of advanced lung adenocarcinoma with SPTBN1-ALK fusion resistant to chemotherapy and radiation therapy in a 69-year-old non-smoking Chinese male 102. Notably, the prognosis was relatively poor 102. The SPTBN1-ALK fusion gene may be a biomarker for refractory cancer and a target for antitumor therapy.

### βII spectrin in head and neck disease

The genetic characteristics of oropharyngeal squamous cell carcinoma (OPSCC) associated with human papillomavirus-positive are currently unknown. The mutation of SPTBN1 is closely related to the occurrence of OPSCC 103, but the specific molecular mechanism of this change is not clear. Another HPV-negative study of OPSCC demonstrated that spectrins are significantly related to poor prognosis 104. Collectively, these studies increase the possibility that SPTBN1 is a useful biomarker in OPSCC, but further research is required to clarify this suggestion.

### βII spectrin in ovarian cancer and breast cancer

In the context of ovarian cancer metastasis and cell migration, little is known about the role of βII spectrin. A recent study indicated that βII spectrin mediates the inhibition of JAK/STAT signaling pathway through suppressor of cytokine signaling 3 (SOCS3), thereby inhibiting cell growth and migration in epithelial ovarian cancer 105. Additionally, βII spectrin has been shown to be involved in the resistance of serous ovarian cancer to cisplatin treatment 106. The research on drug resistance in ovarian cancer showed that the Spectrin αII and βII of cytoskeleton proteins can prevent cisplatin activity and enhance drug resistance 106. Due to the development of drug resistance in ovarian cancer, the molecular mechanism of drug resistance must be elucidated, which is conducive to the best choice of clinical treatment, and the sensitivity of anticancer drugs affects the prognosis of ovarian cancer.

SH3 domain-binding glutamic acid-rich-like protein 2 (SH3BGRL2) has a dual role in breast tumors. The latest research found that SH3BGRL2 inhibits the proliferation of breast cancer cell lines in vitro and the growth of xenograft tumors in vivo 107. Further research found that SH3BGRL2 promotes breast cancer cell migration and invasion by inhibiting the expression of SPTAN1 and SPTBN1. Current research has confirmed that SPTBN1 has changed in breast cancer, but the specific mechanism and role of SPTBN1 in breast cancer need to be further clarified.

### βII spectrin in Lymphoma

The expression of spectrin isoforms in B-cell malignant lymphomas remains unknown. To the best of our knowledge, there has been no comprehensive analysis of spectrin expression in lymphoid malignancies. Spectrin appeared to be downregulated in some cases of classical Hodgkin's lymphoma and nodular lymphocyte-dominant Hodgkin's lymphoma compared to lymphocytes in reactive lymphoplasia 108. Changes in spectrin gene expression in lymphomas may also be related to other gene-silencing mechanisms, such as hypermethylation of DNA, point mutations, and transcriptional repression. There has been little research on the expression of βII spectrin in lymphoma, but they are certainly related. The causal relationship between the specific pattern of spectrin loss and expression in B-cell malignant lymphoma must be confirmed by additional mechanistic studies.

### βII spectrin in uveal melanoma (UVM)

Uveal melanoma (UVM) is the most common adult intraocular malignant tumor with a high metastatic tendency. βII spectrin is an independent prognostic factor for UVM, and its transcription level has high prognostic value and thus may help us better identify UVM or assess UVM progression 109. Therefore, SPTBN1 is an important biomarker for diagnosis.

## The involvement of βII spectrin in other disease

In addition to βII spectrin has an important regulatory role in a variety of cancers, βII spectrin participates in many organ system diseases, such as immune system diseases, nervous system diseases, and circulatory system diseases and so on (Figure 4, Table 2).

### βII spectrin in heart diseases

In the heart, the cytoskeleton is crucial in maintaining the structural integrity of myocardial cells. Likewise, βII spectrin is also critical in maintaining normal development of the heart during embryogenesis 59. The development of severe heart disease, such as congenital arrhythmia, heart failure (HF), and congenital heart disease, is closely related to mutations in the βII spectrin gene.

### Effects of βII spectrin on myocardial excitability

As mentioned above, the integrity of the cytoskeleton associated with cardiomyocytes is an important component to maintain the normal function of cardiomyocytes 110. Membrane-bound ionophore proteins such as channels and transporters have normal coordinated activity and are critical for the normal beating of the heart and the excitability of the heart. Mutations in the proteins that make up the transport device, cytoskeleton proteins and related proteins can cause arrhythmias. Studies have shown that βII spectrin is localized in the intracellular compartment of mouse neonatal cardiomyocytes, βII spectrin and ankyrin B are colocalized in the stripe above the M line 111. The myocardial ankyrin-B-βII spectrin complex is closely related to the inner cell membrane. Elucidating the location, composition, and function of βII spectrin and ankyrin B in myocardial cells and their relationship with cell membrane growth will be important targets for future research. The actin-related molecule βII spectrin is critical for posttranslational targeting and the localization of key membrane proteins in the heart. Mice lacking cardiotype βII spectrin exhibited fatal arrhythmias, abnormal electrical and calcium handling phenotypes, and abnormal expression/localization of cardiac membrane proteins 17, 112. The ankyrin-B/βII spectrin interaction is the basis of key ionophore protein localization and key excitation-contraction coupling protein expression levels. In summary, these findings provide a new mechanism for human excitatory cell disease and reveal a new role for the cardiac cytoskeleton in human cardiovascular disease.

### Effects of βII spectrin on heart failure ( HF ) and remodeling

A previous study compared changes in gene expression in the heart muscle of patients with worsening HF and stable end-stage failure after left ventricular assist device (LVAD) implantation. There was no obvious change in the expression level of the cytoskeleton protein marker βII spectrin among donors at the end of disease in the stable HF group or in the group with worsening HF 113. βII spectrin levels are known to be significantly changed in human cardiovascular disease and animal cardiovascular disease models, and molecular mechanisms related to βII spectrin are emerging targets in the study of HF 114. The critical role of βII spectrin in LVAD remodeling showed that the mechanism of transcriptional mouse cardiac ankyrin repeat protein regulation was altered due to the lack of βII spectrin expression in cardiac cells 115.

These findings indicate that βII spectrin is essential for the normal electrical activity of cardiac muscle cells and cardiac remodeling. Studying the link between this molecule and human heart disease has provided new insights into the biological mechanisms of cardiac muscle cells.

### βII spectrin in myeloproliferative disease (MPD)

MPD is a group of tumorous diseases caused by the continuous clonal proliferation of one or more differentiated mature bone marrow cell lines. A clinical case analysis found that SPTBN1-type III receptor tyrosine kinase (FLT3) fusion was rare in atypical chronic myeloid leukemia (aCML), but abnormal FLT3 was sensitive to immunotherapy, and as a new type of constitutively active tyrosine kinase, it was also sensitive to tyrosine kinase inhibitor therapy 116. Previous studies have reported that the platelet-derived growth factor receptor β (PDGFRB) gene had undergone chromosomal translocation in some patients with atypical MPD. Another clinical case analysis of MPD revealed that the PDGFRB gene encodes a tyrosine kinase receptor. SPTBN1, its newly identified genetic partner, could fuse with PDGFRB to cause its constitutive activation 117. Furthermore, the patient achieved complete molecular remission after treatment with imatinib mesylate 117. This indicates that targeting of the product of the fusion of these genes provides important insight and has research significance for underlying disease pathogenesis, diagnosis, treatment and prognosis. Therefore, fusion and rearrangement of SPTBN1 with other genes are critical to the chemotherapy chosen and clinical prognosis.

### βII spectrin in osteoporosis

Although several genome-wide association studies (GWAS) have confirmed the close association between βII spectrin and osteoporosis 12, 118-122, the mechanism of its role in bone pathophysiology is unknown. Osteoporosis is a disease characterized by low bone mass and microstructural degradation of the bone tissue, resulting in an increased risk of fracture. This increases the possibility that the βII spectrin gene has great importance in regulating bone mass in women. Studies have shown that βII spectrin, a gene associated with osteoporosis, may participate in the pathology of osteoporosis 123. Recently, βII spectrin gene were related to serum periostin levels and genetic variations in bone microstructure 124. As a result, osteoporosis is highly hereditary. Consistent with this finding, several evidence indicate that some single-nucleotide polymorphisms (SNPs) in βII spectrin are associated with osteoporotic fractures 12, 125. These variations indicate osteoporosis susceptibility genes in Han and European populations 126. Bone mineral density (BMD) is a highly genetic indicator of osteoporotic fracture and the most widely used indicator of fracture risk. A genome-wide meta-analysis revealed that βII spectrin were associated with fracture risk 12, 127. These findings reveal the genetic structure and pathophysiology of changes in bone density and fracture susceptibility. In a GWAS of BMD, TGF signal transduction molecules (βII spectrin) were shown to be involved in key biological pathways involving bone and 2p21 (βII spectrin) loci associated with fracture risk 128. Consequently, these findings may provide new insights into underlying genetic assays and jointly identified mechanisms of osteoporosis.

### βII spectrin in nervous system diseases

Previous studies have shown that actin, spectrin, and related molecules form membrane-associated periodic skeletons (MPSs) in axons 129, 130. Trophic deprivation (TD) causes rapid breakdown of MPS 131. βII spectrin may play a role in MPS formation and is a new factor in TD-induced retrograde signals and axonal mutations.

βII spectrin has been identified as one of the components of neocortical Lewy bodies 132. α-Synuclein (a cytosolic neuronal protein) has multiple functions as the main component of Lewy bodies and Lewy neurites. In Parkinson's disease, pathological markers to judge the survival of neuronal cells can be found in Lewy bodies and Lewy neurites. βII spectrin is critical in the normal physiological function of axons. The results of a related study suggest that α-synuclein regulates neurite growth by interacting with cytoskeletal proteins such as βII spectrin 133. Another study found that presynaptic βII spectrin/α-spectrin heterodimers play a vital physiological role in stabilizing synapses and are also involved in regulating the exocytosis of neurotransmitters 134-136. Similarly, in dopaminergic neurons, βII spectrin was essential in regulating growth and could participate in the regulation of neurite growth as well as α-synuclein 133. Lewy body dementia is a disease associated with α-synuclein characterized by neocortical Lewis-associated pathology (LRP). Studies have shown that βII spectrin may be associated with LRP 137. Therefore, βII spectrin may be critical for neurodegenerative diseases.

Neurofibromatosis 2 (NF2) protein (merlin; schwannomin) is involved in the tumorigenesis of some schwannomas and meningiomas, and has an inhibitory effect on the tumor 138. In benign tumors of the human nervous system, mutations in NF2 are common. Many meningiomas and ependymomas do not have NF2 protein, especially almost all schwannomas 139. Changes in the cytoskeleton are early events in the pathogenesis of certain tumor types. Under normal conditions, the interaction between the NF2 and βII spectrin actin binding sites occurs. When NF2 is mutated, the binding is reduced, which changes the normal shape of the cell membrane skeleton and leads to the occurrence of tumors 139.

Collectively, the versatile roles of βII spectrin in regulating axon stability, axonal transport, axonal Ranvier node assembly, neurite growth, neurodegenerative diseases and benign tumors of the nervous system have been demonstrated in increasing numbers of studies.

### βII spectrin in immune system diseases

Macrophages are significant in HIV-1 infection and serve as the main target of HIV-1. The knockdown of βII spectrin severely damaged the cytoskeleton structure, so βII spectrin is necessary to maintain the macrophage actin cytoskeleton structure. Interleukin 27 (IL-27) is a pleiotropic cytokine that is crucial and has many functions in the immune system 140. Studies have shown that monocytes differentiate into macrophages after their activation by IL-27 and that macrophages are not sensitive to HIV-1 infection. In monocytes, βII spectrin expression was not detected, but it was found to be significantly upregulated during macrophage differentiation, and IL-27 caused a decrease in the sensitivity of macrophages to HIV-1 infection by inhibiting βII spectrin expression. In contrast, overexpression of βII spectrin increases sensitivity. Specifically, IL-27 downregulated βII spectrin expression through TAK-1-mediated mitogen-activated protein kinase (MAPK) signaling pathways. βII spectrin can act as an intracellular adaptor of HIV-1 viral particles after they enter macrophages. The results of this study have demonstrated that βII spectrin is an essential host factor for macrophage HIV-1 infection. Of course, the exact role of βII spectrin on macrophages throughout the life cycle of HIV-1 infection needs further study.

### βII spectrin in eye diseases

Intraocular pressure (IOP) is one of the major risk factors for glaucoma. The study of genetic factors closely related to IOP can simultaneously provide new insights into the genetic mechanism of glaucoma and new directions and methods for the clinical diagnosis and treatment of glaucoma. A GWAS showed that βII spectrin expression is an eye-related phenotype 141 that is also closely related to IOP 142. The relationship between βII spectrin and IOP revealed in this study not only increased our understanding of biomarkers in IOP but also provided important insights into the genetic markers of glaucoma and will provide future genetic screening and treatment methods for glaucoma related to IOP. Furthermore, new molecular markers may be identified. Primary congenital glaucoma (GLC3) is a hereditary eye disorder that may be associated with βII spectrin 143. Studies have suggested that βII spectrin can be used as a biomarker with diagnostic value in some eye diseases. Likewise, it also has significant prognostic value.

### βII spectrin in stem cell disease

Cancer stem cells (CSCs) in HCC have functions that trigger tumorigenesis, metastasis and drug resistance. The main reason for the induction of HCC is the alteration of key pathways that inhibit tumorigenesis in its stem cells. However, the molecular mechanism by which metastatic stem cells are produced is unclear. The authors speculate that the main reason for the transformation of genetically defective LSCs into highly metastatic liver cancer cells in precancerous liver tissue is chronic inflammation 33. This was demonstrated in a model of chronic inflammation in βII spectrin-expressing (βII spectrin^+/-^) mice, where were highly tumorigenic and metastatic and positive for Epithelial-mesenchymal-transition (EMT) 33. In CD133^+^ LSCs, this process involves constitutive activation of the transforming growth factor-β-activated kinase 1 (TAK1)- Nuclear factor kappa -light-chain-enhancer of activated B cells (NFκB) signal by an inflammatory program mediated by IL6 33. Another study demonstrated that IL6 can activate STAT3, thereby inducing the HCC process (Figure 3) 144. The TGF-β signaling pathway is also involved in the above process, activated kinase 1 (TAK1; MAP3K7 mitogen-activated protein kinase kinase kinase 7 (MAP3K7)), leading to increased production of metastatic CSCs 33. This study showed for the first time that βII spectrin inhibits the development of metastatic clones and CSCs through the TGF-β pathway. In short, the molecular interaction between βII spectrin and IL6 as a mechanism for the development of CSCs provides new directions and insights. Another event in the development of liver cancer that cannot be ignored is the dedifferentiation of liver cells. Related studies have shown that βII spectrin plays a role in promoting differentiation and inhibiting growth in vitro during the differentiation of LSCs 68. In addition, βII spectrin can inhibit the transcriptional activity of liver CSC markers through β-catenin. In conclusion, βII spectrin plays a role in promoting the differentiation of liver CSCs, leading to the inhibition of CSC function in liver cancer-initiating cells. Restoration of βII spectrin expression may become a new method to prevent and treat liver cancer.

Beckwith-Wiedemann syndrome (BWS) is an overgrowth of stem cells 145, and one of its causes is the loss of βII spectrin 93. Epigenetic silencing of the SMAD3/4 scaffold protein βII spectrin may be the causative factor of BWS 146. Two heterozygous βII spectrin^+/-^/Smad3^+/-^ mice developed a variety of tumors due to defective TGF-β signaling, and the phenotypes of these tumors were highly similar to those in BWS patients 146. Nonetheless, the specific molecular mechanism of βII spectrin in the disease and whether the recovery of βII spectrin has a therapeutic effect on BWS need more explicit research. In conclusion, βII spectrin has important potential for molecular therapy in the future.

## The potential of βII spectrin (SPTBN1) as a therapeutic target

βII spectrin is a widely expressed protein with multiple functions. Its mutations and disorders affect the normal growth and development of organs 59. βII spectrin contains many phosphorylation sites and can be used as a potential modulator of the interaction between proteins. Thr-2159 specifically exists in βIIΣ2-spectrin, and its phosphorylation reduces the affinity of the C-terminal region of βIIΣ2 and the N-terminal region of αII subtype. In in vitro experiments, although the affinity of mutant T2159E with αII-spectrin is reduced, it does not prevent the remodeling of cell shape and the growth of neurites 24. Interestingly, during the growth of neurites, βIIΣ2-spectrin with mutation T2159A can effectively inhibit growth 24. Therefore, T2159 site can be used as a potential therapeutic target in related organ development. Moreover, βII spectrin was also demonstrated to be essential for heart development. In SPTBN1 gene conditional knockout mice, the embryonic heart development was affected and the heart structure had obvious defects 59. Especially after E14.5, the ventricular wall of the mutant embryo heart cannot be thickened 59. Collectively, all these evidence indicated that mutations and deletions of βII spectrin cause developmental abnormalities and thus affect organ functions. And the expression level of βII spectrin has important diagnostic significance in congenital dysplasia, and βII spectrin may be a therapeutic target for reversing developmental abnormalities. Additionally, βII spectrin^-/ -^ mice and smad2^+/-^/smad3 ^+/-^ mutant mice showed similar phenotypes 14, 147. The mice died in the second trimester of pregnancy because of developmental defects in the gastrointestinal tract, liver, heart and nerves 14. Notably, many phenotypic characteristics of βII spectrin^-/-^ mice are similar to those of BWS patients, such as visceral hypertrophy, megaglossia, abnormal ear folds, etc 93, 146. Therefore, the βII spectrin mutation may be involved in the mechanism of human BWS. It also includes several malignant tumors that occur within a few months, such as liver cancer, stomach cancer, bowel cancer, pancreatic cancer, kidney cancer, and adrenal adenocarcinoma 32, 93, 148. Moreover, abnormal angiogenesis exists in HCC from βII spectrin^-/-^ mice, indicating its role as an angiogenesis regulator that inhibits HCC 31. In short, the in-depth study targeting βII spectrin is of great value for early detection of these diseases, formulating new treatment strategies and determining biomarkers of related diseases.

To date, the changes of βII spectrin in clinical diseases cannot be ignored. As mentioned above, previous studies have also demonstrated that SPTBN1 was down-regulated in a variety of cancers, such as hepatocellular carcinoma, gastrointestinal tumors and lung cancer 10, 30, 69. At the same time, its expression level is closely related to the patient's prognosis, such as hepatocellular carcinoma, pancreatic cancer and lung cancer 61, 66, 72, 73, 102. This increases the possibility of SPTBN1 as a biomarker for cancer diagnosis and prognosis. As well known, the choice of cisplatin could significantly improve the prognosis of patients with ovarian cancer. In the study of clinical cases, it was found that αII spectrin and βII spectrin can prevent the activity of cisplatin to promote drug resistance 106. For cases where drug resistance may occur, if the treatment drugs for refractory cases can be determined in advance, then the side effects of patients can be greatly reduced, the treatment effect and prognosis of patients can be improved. Therefore, drugs that target the βII spectrin resistance mechanism will improve the effectiveness of chemotherapy in refractory cases. Moreover, the SPTBN1-ALK fusion gene found in lung adenocarcinoma may become a potential biomarker for refractory cancer, and the prognosis is relatively poor 102. Therefore, in the selection of treatment methods, the new SPTBN1-ALK fusion gene may be a potential target for anti-cancer therapy.

In conclusion, βII spectrin is critically involved in the development and occurrence of diseases, especially neurodevelopment and cancer progression. Future research on βII spectrin as a diagnostic and therapeutic target will provide new options for the treatment of a variety of human diseases.

## Conclusion and outlook

As mentioned above, βII spectrin is indispensable for normal growth and development of organs. Deletion of βII spectrin resulted in changes in various cellular functions, such as proliferation, differentiation, apoptosis, the cell cycle and adhesion. Additionally, βII spectrin is associated with DNA damage repair and EMT. In this review, we have summarized data showing that βII spectrin dysfunction or deficiency can lead to arrhythmia, HF, neurodegeneration, osteoporosis and embryonic death. We also reviewed what is currently known about the role of βII spectrin in CSCs and immune disease. Notably, βII spectrin is involved in the infection of human macrophages with HIV-1. This is an exciting discovery and provides new insights into the treatment of HIV. Nonetheless, the signaling pathways and molecular mechanisms involved in the occurrence and development of the above diseases need to be further studied.

Strikingly, a recently published study described that βII spectrin is critical for CRC through TGF-β signaling. It suggested that βII spectrin has great potential in exploring the genesis and development of CRC and as a therapeutic agent. In addition to the vital role of βII spectrin in CRC, low βII spectrin expression in patients with pancreatic cancer indicates a poor prognosis and shorter survival time. Notably, βII spectrin plays a dual role in liver cancer. Several related pieces of evidence have demonstrated that the expression of βII spectrin is significantly reduced in most human HCC cases. Nevertheless, βII spectrin was overexpressed in a highly metastatic HCC cell line. Interestingly, overexpression of βII spectrin in liver cancer accelerated the invasion of liver cancer cells, but the mechanism of this process requires further research. βII spectrin has been shown to be involved in the resistance of serous ovarian cancer to cisplatin treatment, and the fusion of SPTBN1 with other genes also causes resistance. Due to the development of drug resistance, its molecular mechanism must be elucidated, which will be conducive to selecting the best clinical treatment. These results suggest that targeting of the fusion product of these genes has important insight and research significance into underlying disease pathogenesis, diagnosis, treatment and prognosis.

Altogether, there is growing evidence that βII spectrin has not only predictive but also prognostic value. Therefore, an in-depth understanding of the biology of βII spectrin will help develop new clinical treatment strategies. This review will advance future study of the role of membrane skeletal proteins in cancer and other diseases, particularly regarding the close intertwining of their functions and structural characteristics; provide molecular mechanisms and directions for the clinical diagnosis and treatment of diseases; and provide evidence and basic insight into potential future research areas, especially neurological diseases and cancers.

## Figures and Tables

**Table 1:** βII spectrin changes in cancer

Tumor Type	Changes in βII spectrin	References
Hepatocellular carcinoma	βII spectrin downregulation βII spectrin upregulation	^32, 61, 62, 74, 75, 79-81^ ^81^
pancreatic cancer	βII spectrin downregulation miR-145 downgrade βII spectrin	^73^ ^70^
Colorectal cancer (CRC)	SPTBN1-ALK fusion protein: potential targets for chemotherapy	^96^
Esophageal cancer	βII spectrin downregulation	^71^
Lung cancer	SPTBN1-ALK fusion: resistant to chemotherapy and radiation therapy	^102^
Follicular lymphoma	Loss of βII spectrin	^108^
Uveal melanoma (UVM)	Alterations in SPTBN1 is significantly associated with worse prognosis	^109^
Ovarian cancer	βII spectrin can prevent cisplatin activity and enhance drug resistance	^106^
Oropharyngeal squamous cell carcinomas (OPSCC)	βII spectrin is mutated	^103^
	Tumor expressed spectrin were more likely to die at any given time when compared with those without spectrin	^104^
Atypical chronic myeloid leukemia (aCML)	SPTBN1-FLT3 fusion: chemotherapy target	^116^
Gastric cancer	Loss of ELF	^101^

**Table 2:** βII spectrin in other diseases

Disease Type	Changes in βII spectrin	References
Arrhythmia	βII spectrin downregulation	^17, 112^
Heart failure (HF)	No change	^113^
Myeloproliferative disease (MPD)	SPTBN1-PDGFRB: chemotherapy target	^117^
Osteoporosis	Variation in SPTBN1 expression	^12, 118-121, 123-128, 149^
Neurodegenerative diseases	βII spectrin mutation	^137^
HIV	βII spectrin upregulation	^140^
Primary congenital glaucoma (GLC3)	Related candidate gene	^143^
Beckwith-Wiedemann syndrome (BWS)	Loss of βII spectrin	^93^

## Abbreviations

Akt: Protein kinase B
BMD: Bone mineral density
CDK4: Cyclin-dependent kinase 4
CEACAM1-4L: Carcinoembryonic antigen-related cell adhesion molecule 1
CHL1: Close homolog of L1
CSC: Cancer stem cell(s)
EpCAM: Epithelial cell adhesion molecule
EOC: Epithelial ovarian cancer
EMT: Epithelial-mesenchymal-transition
ESP: Endometrial side population
FLT3: Type III receptor tyrosine kinase
GWAS: Genome-wide association studies
GSK3: Glycogen synthase kinase 3α/β
HCs: Hair cells
HCC: Hepatocellular carcinoma
IL6: Interleukin 6
LSCs: Liver stem cells
LRP6: Low density lipoprotein receptor-related protein 6
LVAD: Left ventricular assist device
MPD: Myeloproliferative disease
MPSs: Membrane-associated periodic skeletons
MAPK: Mitogen-activated protein kinase
NFκB: Nuclear factor kappa -light-chain-enhancer of activated B cells
OPSCC: Oropharyngeal squamous cell carcinoma
PI3K: Phosphoinositide 3-kinase
PMSs: Periodic membrane skeletons
PNS: Peripheral nervous system
PDGFRB: Platelet-derived growth factor receptor β
Rb: Retinoblastoma protein
STAT3: Transcription 3
TGFβ: Transforming growth factor β
TLR4: Toll-like receptor 4
TICs: Tumor-initiating stem-like cells
YAP: Yes-associated protein
